# Surface-Adsorbed Contaminants Mediate the Importance of Chemotaxis and Haptotaxis for Bacterial Transport Through Soils

**DOI:** 10.3389/fmicb.2019.02691

**Published:** 2019-11-26

**Authors:** Liqiong Yang, Xijuan Chen, Xiangfeng Zeng, Mark Radosevich, Steven Ripp, Jie Zhuang, Gary S. Sayler

**Affiliations:** ^1^Key Laboratory of Pollution Ecology and Environmental Engineering, Institute of Applied Ecology, Chinese Academy of Sciences, Shenyang, China; ^2^College of Resources and Environment, University of Chinese Academy of Sciences, Beijing, China; ^3^Department of Biosystems Engineering and Soil Science, University of Tennessee, Knoxville, TN, United States; ^4^Department of Microbiology, Center for Environmental Biotechnology, University of Tennessee, Knoxville, TN, United States

**Keywords:** bacterial transport, naphthalene, saturated flow, chemotaxis, haptotaxis

## Abstract

Chemotaxis and haptotaxis are important biological mechanisms that influence microbial movement toward concentrated chemoattractants in mobile liquids and along immobile surfaces, respectively. This study investigated their coupled effect, as induced by naphthalene (10 mg L^−1^), on the transport and retention of two pollutant-degrading bacteria, *Pseudomonas fluorescens* 5RL (*Pf*5RL) and *Pseudomonas stutzeri* DQ1 (*Ps*DQ1), in quartz sand and natural soil. The results demonstrated that *Ps*DQ1 was not chemotactic, whereas *Pf*5RL was chemotactic at 25°C but not at 4°C due to the restricted movement. In a quartz sand column, haptotaxis did not play a role in increasing the transport of *Pf*5RL as compared with chemotaxis. Compared with a naphthalene-free soil column, *Pf*5RL broke through naphthalene-presaturated soil columns to reach a stable effluent concentration 0.5 pore volumes earlier due to advective chemotaxis occurring behind the plume front in the bulk solution. *Pf*5RL also demonstrated greater retention (e.g., a doubled rate of attachment and a one-third smaller breakthrough percentage) due to along-surface haptotaxis and near-surface chemotaxis occurring in less mobile water near the soil surface. However, both chemotaxis and haptotaxis were weakened when *Pf*5RL co-transported with naphthalene due to reduced adsorption of naphthalene on the soil. This study suggests that surface adsorption of naphthalene can mediate the relative importance of advective chemotaxis (facilitating initial breakthrough), near-surface chemotaxis (increasing bacterial collision), and haptotaxis (increasing bacterial residence time).

## Introduction

Movement of certain bacteria, such as *Pseudomonas fluorescens*, is subject to chemical gradients (Law and Aitken, 2005; Singh and Olson, 2012; Yan et al., 2014; Adadevoh et al., 2018), as demonstrated when they move toward chemoattractants (e.g., glucose) or away from repellants (e.g., phenol) (Harwood et al., 1989; Roush et al., 2006; Pham and Parkinson, 2011). Directional motion toward chemoattractants along a concentration gradient in aqueous phase is called chemotaxis, while migration toward concentrated chemoattractant along an immobilized solid surface is called haptotaxis (Carter, 1965, 1967; Ricoult et al., 2015; Roy et al., 2017). Both rely on chemical signals that are generated and heterogeneously distributed in the microenvironment as a result of complex biochemical phenomena. Chemotaxis can influence many microbial processes, such as those related to contaminant bioremediation (Marx and Atiken, 2000; Pandey and Jain, 2002; Wang et al., 2016), biofilm formation (Pratt and Kolter, 1998; Ahmed et al., 2010), nutrient cycling in the ocean (Jackson, 1989; Blackburn et al., 1998; Azam and Long, 2001; Stocker et al., 2008), disease pathogenesis (Ottemann and Lowenthal, 2002), wound healing and cancer (Keenan and Folch, 2007), and cellular differentiation and growth (Kim et al., 2015). Thus far, chemically guided movement of bacteria in aqueous systems has been well documented (Harwood et al., 1989; Frymier et al., 1995). Unfortunately, fewer studies have been conducted in heterogeneous soil systems, and little attention has been given to the role of soil surfaces, which greatly influence near-surface chemotaxis and along-surface haptotaxis of bacteria.

In the presence of chemoeffectors (e.g., naphthalene, fumarate, acetate), chemotactic bacteria decrease the frequency of flagella rotational change in order to bias their movement preferentially in the direction of an increasing attractant concentration (Harwood et al., 1989). Bacteria can even move toward the attractant in bulk when the gradient chemoattractants are in a direction perpendicular to the convective flow (Lanning et al., 2008). Bacteria with flagella can swim through aqueous media at 1.7–3.5 m day^−1^ (Harwood et al., 1989; Frymier et al., 1995), making chemotaxis an important mechanism influencing bacterial transport at low flow rates or in less permeable media (Witt et al., 1999; Olson et al., 2004; Wang and Ford, 2009; Wang et al., 2014). It was reported that chemotaxis could improve bioremediation by enhancing microbial accessibility to pollutants particularly in soil environments of low hydraulic permeability (Taraboletti et al., 1987; Harwood et al., 1990; Harms, 1996; Marx and Atiken, 2000; Wang et al., 2016). However, it might be inaccurate to simply attribute all impacts caused by chemoattractant gradients to advective chemotaxis in soil pores if soils strongly adsorb the chemoattractant to establish concentration gradients in less mobile water near soil surfaces and along the rough, heterogeneous soil surfaces. Near-surface chemotaxis might induce bacteria to collide with soil surfaces, thus increasing bacterial attachment efficiency. Later, the chemoattractant gradients along the soil surface could trigger haptotaxis to increase the residence time of the attached bacteria. Theoretically, the influences of near-surface chemotaxis and haptotaxis might be much stronger in porous media that have adsorbed more chemoattractants (e.g., naphthalene or glucose). However, this assumption has not been examined, considerably confusing the understanding of the dependence of chemotaxis on soil properties and chemoattractant distribution among mobile, less mobile, and immobile phases.

As a representative of polycyclic aromatic hydrocarbons (PAHs), naphthalene is one of the most prevalent groundwater contaminants at sites contaminated with PAHs (Mihelcic and Luthy, 1991; Ghoshal et al., 1996; Marx and Aitken, 1999). Although naphthalene-induced bacterial migration has been well studied as a chemotactic phenomenon, the majority of studies were conducted in homogeneous model porous media by placing pure crystalline naphthalene somewhere in the porous media to observe the induced change in bacterial deposition (Marx and Atiken, 2000; Law and Aitken, 2003; Velasco-Casal et al., 2008; Adadevoh et al., 2015). This strategy creates macroscopic concentration gradients for straightforward observation of chemotactic movement in the liquid phase. However, naphthalene does not always exist as a pure crystalline solid in natural environments. It can exist in the form of vapor and enter soil and water with rainfall and dust precipitation, as a component of oily sewage, or as a constituent of liquid fuels that release into the environment. Aqueous phase naphthalene may permeate aquifers together with bacteria, or bacteria may invade the naphthalene-polluted area, where naphthalene is adsorbed in micropores of heterogeneous subsurface sediments. These conditions may also occur with other PAHs or contaminants. These processes can create interfacial concentration gradients in the less mobile surface pore-retained water near soil surfaces and along the immobile surfaces of heterogeneous soils. The former gradient causes near-surface chemotaxis to increase bacterial collision efficiency, and the latter triggers haptotaxis to redistribute the colliding bacteria to naphthalene-rich sites (such as organic matter sites) resulting in extended residence time. Therefore, aqueous naphthalene might facilitate bacterial migration through advective chemotaxis in the bulk solution, while surface-bound naphthalene might reduce bacterial migration through near-surface chemotaxis and/or along-surface haptotaxis (see Supplementary Figure S1 for a conceptual representation of these processes). A theoretical assumption is that soil heterogeneity and associated non-ideal flow could alter the relative importance of these mechanisms. This study aimed to develop an understanding of the surface-mediated effects of chemotaxis and haptotaxis on the transport and retention of chemotactic bacteria in natural soils.

## Materials and Methods

### Porous Materials

A model quartz sand and a natural soil were used for the column experiments. The quartz sand was obtained from Tianjin Kemiou Chemical Reagent Co. Ltd. (Tianjin, China) and had a coarse texture with a mass median diameter (d_50_) of 700 μm. Prior to the experiments, the sand was washed with HCl (10 mM) and then NaOH (10 mM) solutions to remove suspended impurities and finally rinsed with deionized water. The natural soil was a chestnut soil (sandy, mixed, active, mesic Typic Dystrudepts) obtained from Inner Mongolia, China. It had a coarse texture with a d_50_ value of 500 μm and contained 23.5% coarse sand (2–0.25 mm), 58.3% fine sand (0.25–0.05 mm), 2.5% silt (0.05–0.005 mm), and 15.7% clay (<0.005 mm). The soil contained a significant amount of organic matter (1.0%) relative to the negligible organic matter present in the quartz sand.

### Adsorption of Naphthalene

We conducted batch experiments in three replicates to determine the kinetic and equilibrium behaviors of naphthalene adsorption on the sand and the soil. The adsorption kinetic experiments were carried out by mixing 5.0 g of quartz sand or chestnut soil with 50 ml of a naphthalene solution (10 mg L^−1^) in a flask. The whole system was sterilized to eliminate the effect of microbial degradation. The flask was sealed and shaken on a rotary shaker for 24 h at 160 rpm and 25°C. During the shaking, the suspension was sampled every 1 h and filtered through a 0.45-μm membrane for naphthalene analysis using HPLC (Agilent, 1260LC). Isocratic (80% methanol and 20% deionized H_2_O) separation was accomplished on a C-18 column with detection at 254 nm and at a flow rate of 1 ml min^−1^. The detection limit of naphthalene was 0.04 mg L^−1^ in this study. The adsorption kinetics of naphthalene on the sand and soil were determined using the pseudo-first order model (Lagergren, 1898; Osagie and Owabor, 2015):

(1) $$Q_{t}\;=\;Q_{e}\;\left(1-e^{-kt}\right)$$

where *Q*_t_ (mg kg^−1^) and *Q*_e_ (mg kg^−1^) are the adsorbed capacities at time *t* (h) and at equilibrium, respectively, and *k* (h^−1^) is the rate constant.

The adsorption equilibrium experiments were carried out in a series of 100-ml vials, which contained 5.0 g of quartz sand or chestnut soil (including no-solid controls) and 50 ml of naphthalene solution at the concentrations of 1, 5, 10, 15, 20, and 25 mg L^−1^, on a rotary shaker at 25°C for 24 h (pre-determined to be sufficient to reach equilibrium). The whole system was sterilized to avoid the effect of microbial degradation. The suspension was then centrifuged at 2,012 g for 10 min and filtered through a 0.45-μm membrane prior to analysis using HPLC (Agilent, 1260LC) as described above. Naphthalene partitioning to the sand or soil was calculated using the following equation:

(2) $$Q_{e}\;=\;k_{d}C_{e}$$

where *Q_e_* (mg kg^−1^) is the specific equilibrium concentration of naphthalene adsorbed on solid phase, *C_e_* (mg L^−1^) is the equilibrium concentration of naphthalene in the aqueous phase, and *k_d_* (L kg^−1^) is the partition coefficient of naphthalene between the aqueous phase and solid phase.

### Bacteria and Culture Conditions

This study was performed using two motile bacterial strains, *Pseudomonas fluorescens* 5RL (*Pf*5RL) and *Pseudomonas stutzeri* DQ1 (*Ps*DQ1). *Pf*5RL is chemotactic to naphthalene (King et al., 1990) and was obtained from the Center for Environmental Biotechnology, University of Tennessee, Knoxville, USA. *Ps*DQ1 is physiochemically similar (in size, zeta potential, and contact angle) to *Pf*5RL but is not chemotactic to naphthalene (Zhao et al., 2015) and was obtained from the Institute of Applied Ecology, Chinese Academy of Sciences, Shenyang, China. Both strains were motile by polar flagellar rotation, negatively charged, exhibiting similar zeta potential. *Pf*5RL carries a plasmid in which metabolic genes for salicylate degradation have been replaced with *lux* genes responsible for bioluminescence. Thus, salicylate induces bioluminescence but is not degraded, and within limits, the resulting light is proportional to salicylate concentrations (Oates et al., 2005). *Ps*DQ1 is able to use naphthalene as a sole source of carbon and energy. Both strains were individually cultured in sterile 250-ml baffled shake flasks containing 100 ml of yeast extract/peptone/glucose (YEPG) media (Heitzer et al., 1992). A stock filter-sterilized (0.45 μm) naphthalene solution was prepared *via* the dissolution of crystalline naphthalene, which was then added at 10 mg L^−1^ to induce the chemotactic response of *Pf*5RL. Flasks were incubated in a rotary shaker (HZQ-X160) at 160 rpm and 30°C. Tetracycline (CAS 60-54-8) was added to the *Pf*5RL culture at a concentration of 10 mg L^−1^. Cultures were grown to stationary phase (optical density of 1.0 at a wavelength of 600 nm), centrifuged at 1,000 *g* for 10 min, and re-suspended in 500 ml of 10% random motility buffer (RMB) (background solution) (Adadevoh et al., 2015). Cell-surface hydrophobicity was derived from the contact angles (*θ_w_*) of water drops on the bacterial lawns using a goniometer microscope (Krüss GmbH, Germany). A fluorescence microscope (NIKON Ti-E, Japan) was used for measurement of cell length and width (*n* ≥ 50 cells for each individual experiment). The zeta potential of cells was determined using a laser particle analyzer (Brookhaven, 90Plus Zeta) (*n* ≥ 50). All measurements were made using cultures at stationary growth phase.

### Chemotaxis Assay

Chemotaxis of *Pf*5RL to naphthalene was verified *via* capillary testing following standard methods (Adler and Dahl, 1967; Adler, 1973; Law and Aitken, 2003). Briefly, early stationary phase cells (corresponding to 10^6^ cells ml^−1^) were harvested, centrifuged, and re-suspended in 10% RMB. A U-shaped tube was laid between a microscope slide and a glass coverslip to form a small chamber, which was subsequently filled with deaerated bacterial suspension. A capillary tube, heat-sealed at one end, containing the chemoattractant solution (RMB supplemented with 10 mg L^−1^ naphthalene), was inserted into the bacterial suspension at its open end. RMB-lacking naphthalene was used as a control. The chambers were incubated for 4 h at 25°C and then the bacterial concentration in the capillaries was determined by quantifying colony-forming units (CFUs) on YEPG solid media.

### Bacterial Movement Mediated by Naphthalene Distribution

*Pf*5RL radiates visible light when they degrade naphthalene into salicylate. We thus used the bioluminescent imaging technique (IVIS Spectrum, Perkin Elmer) to examine the effect of surface-bound naphthalene on bacterial attachment and detachment in real time. A short glass column (0.5 cm in depth and 3.5 cm in diameter) was used to mimic a segment of the long column used in the transport experiments while minimizing the occurrence of depth profile of bacterial retention in the porous media. The short column was autoclaved and then packed with the quartz sand and the chestnut soil half-by-half across the cross-sectional area. After the column was saturated with naphthalene, the cross-section was imaged. Then, 10 pore volumes of *Pf*5RL suspension were introduced into the column at the same pore velocity as used for the transport experiments before another image was taken. The column was then eluted with a sterilized background solution (10% RMB) until there was no naphthalene in the effluent, and then four images were taken immediately, 1, 2, and 3 h after the termination of elution, respectively. Finally, nine pore volumes of naphthalene-free *Pf*5RL were re-injected into the column to take a final image. The bioluminescent images reflect the spatiotemporal distribution of *Pf*5RL in response to surface-bound naphthalene.

### Haptotaxis Assay

The colloids (<2 μm) were extracted from the chestnut soil and had a specific surface area of 23 m^2^ g^−1^ (calculated from N_2_ adsorption isotherm). The sterilized colloids were pre-equilibrated with low (10 mg L^−1^) and high (20 mg L^−1^) concentrations of naphthalene solutions for 12 h at 25°C, respectively, and then air-dried and ground before they were uniformly put on the glass slide as a thin layer (0.2 mm). The water content of the air-dried colloids was 15.1 g kg^−1^, which was equivalent to a water film thickness of 0.66 nm or 2–3 layers of water molecules, assuming a uniform distribution of water on the colloid surfaces. The colloid layer was divided into three parallel strips: no naphthalene adsorption (N0), low naphthalene adsorption (N1, 47 mg kg^−1^), and high naphthalene adsorption (N2, 90 mg kg^−1^). The assay began by putting a drop of *Pf*5RL suspension on the boundary line between N0 and N1. Images were acquired after 1, 10, 60, 120, 300, and 600 min using a bioluminescent imaging technique (IVIS Spectrum, Perkin Elmer). To check whether *Pf*5RL moved into the N0 strip, we added 0.1 ml of naphthalene solution (10 mg L^−1^) to the dry N0 strip at 600 min, and another image was taken at 610 or 630 min.

### Column Experiments

The column assembly was comprised of a glass chromatography column (3.5 cm in diameter, 10 cm in length) containing sterilized quartz sand or natural soil at a porosity of ~0.36. The column was dry-packed in 1-cm increments with stirring and tapping. After packing, high-pressure CO_2_ gas was introduced upward into the column at a low flow rate for 3 h to displace trapped air. The packed column was then flushed with sterilized background solution (10% RMB) at a pore velocity of ~12.5 cm h^−1^ for 20 pore volumes (~15 h) to saturate the column. The column experiments were performed at two temperatures (25 and 4°C) in two replicates under saturated steady flow conditions at the same pore velocity as used for the flush. Each experiment used freshly packed columns, with an input bacterial concentration of ~4 × 10^8^ cells ml^−1^, as determined by viable plate counts on YEPG media supplemented with tetracycline at 10 mg L^−1^. Bacterial cultures were mixed with NaBr at 50 mg L^−1^ as a conservative tracer for quantifying dispersivity and hydrodynamic conditions of the packed columns. Naphthalene (10 mg L^−1^) and/or bacteria were injected into the packed columns for 10 pore volumes in four different input modes: Input Mode 1—injection of bacteria only (no chemotaxis and no haptotaxis in both sand and soil), Input Mode 2—co-injection of bacteria and naphthalene (sand: weak near-surface chemotaxis and haptotaxis; soil: strong near-surface chemotaxis and haptotaxis), Input Mode 3—injection of naphthalene immediately followed by injection of bacteria (sand: only advective chemotaxis; soil: strong near-surface chemotaxis and haptotaxis), and Input Mode 4—injection of naphthalene followed by a flush with background solution and then injection of bacteria (sand: not applicable; soil: only near-surface chemotaxis and haptotaxis). These input modes created different co-existence situations of pollutants and microorganisms and different distribution ratios of naphthalene between the immobile solid phase and mobile liquid phase within the columns, thereby allowing evaluation of the relative contributions of chemotaxis and haptotaxis to bacterial transport. After collecting a given number of effluent samples, the columns were flushed with the background solution for 10 pore volumes to examine the detachability of retained bacteria and to estimate the total mass recovery of injected bacteria. The effluent samples were collected through a fraction collector every one-fourth pore volume. Each effluent sample was divided into three parts. One part was used to analyze bromide concentration using ion chromatography (DIONEX, ICS-5000), another part was used to measure cell concentration by plate counting, and a third part was used to determine the concentration of naphthalene using HPLC (Agilent, 1260LC). The cell measurement was performed twice for the same effluent sample to obtain a mean value, and then the two mean values of the replicated columns for the same pore volume were averaged again to obtain a final concentration and an error bar for plotting. The hourly measurements of the input bacterial and naphthalene concentrations during the entire experiment indicated that the bacterial suspension remained stable. The breakthrough curves were generated by plotting the relative effluent concentration (C/C_0_) as a function of pore volumes of injected suspension or solution. The C/C_0_ is the ratio of effluent bacterial concentration to influent bacterial concentration, and the pore volume represents the number of replacement of the liquid in the column by the injected liquid. The ascending phase of the transport curve is mainly related to adsorption or attachment, the descending phase is mainly related to desorption or detachment, and the stabilization phase is related to the capacity of adsorption or attachment. One-way analysis of covariance (ANCOVA) (PROC GLM, SAS Institute, 1999) was used to determine whether the breakthrough curves of replicate experiments differed significantly (*p* < 0.05).

### Transport Modeling

Breakthrough curves of bromide, naphthalene, and bacteria were fitted to a one-dimensional advection-dispersion equation (ADE) to obtain transport parameters for quantitative analysis on the chemotactic responses of bacterial transport to naphthalene distribution in the columns under different flow modes. Hydrus-1D software was used to fit the bromide data to a one-dimensional classical ADE (Firouzi et al., 2015) as given below:

(3) $$\frac{\partial C}{\partial t}\;=\;D\frac{\partial^{2}C}{\partial z^{2}}-v\frac{\partial C}{\partial z}$$

where *C* (mg L^−1^) is the bromide concentration in liquid phase, *t* (h) is the time, *v* (cm h^−1^) is the average pore water velocity, *D* (cm^2^ h^−1^) is the dispersion coefficient, and *z* (cm) is the travel distance. The bacterial and naphthalene data were modeled using a modified ADE form that includes two-site kinetic attachment-detachment processes. The total bacterial or naphthalene mass balance equation is defined as:

(4) $$\frac{\partial C}{\partial t}+\frac{\rho }{\theta }\frac{\partial S_{1}}{\partial t}+\frac{\rho }{\theta }\frac{\partial S_{2}}{\partial t}\;=\;D\frac{\partial^{2}C}{\partial z^{2}}-v\frac{\partial C}{\partial z}-\mu_{l}C$$

where *S_1_* (N g^−1^, with N representing the number of bacteria, or mg kg^−1^) and *S_2_* (N g^−1^ or mg kg^−1^) are the solid phase concentrations of bacteria or naphthalene associated with attachment sites 1 and 2, respectively; *μ_l_* denotes bacterial decay in the liquid phase; and *D* (cm^2^ h^−1^) is obtained from the fitting of bromide data. Subgroups to Eq. (4) are Eqs. (5) and (6):

(5) $$\frac{\rho }{\theta }\frac{\partial S_{1}}{\partial t}\;=\;k_{att1}C-k_{\det1}S_{1}\frac{\rho }{\theta }-\mu_{s}\rho S_{1}$$

(6) $$\frac{\rho }{\theta }\frac{\partial S_{2}}{\partial t}\;=\;k_{att2}C-k_{\det2}S_{2}\frac{\rho }{\theta }-\mu_{s}\rho S_{2}$$

where *θ* (cm^3^ cm^−3^) is the volumetric water content; *ρ* (g cm^−3^) is the soil bulk density; *C* (N ml^−3^ or mg L^−1^) is the bacterial or naphthalene concentration in the liquid phase; *k_att_* (h^−1^) is the first-order attachment coefficient; *k_det_* (h^−1^) is the first-order detachment coefficient; subscripts 1 and 2 refer to the fast and slow kinetic sites, respectively; and *μ_l_* (h^−1^) and *μ_s_* (h^−1^) represent inactivation/degradation processes in the liquid and solid phases, respectively.

## Results

### Adsorption of Naphthalene

**Figure 1:**
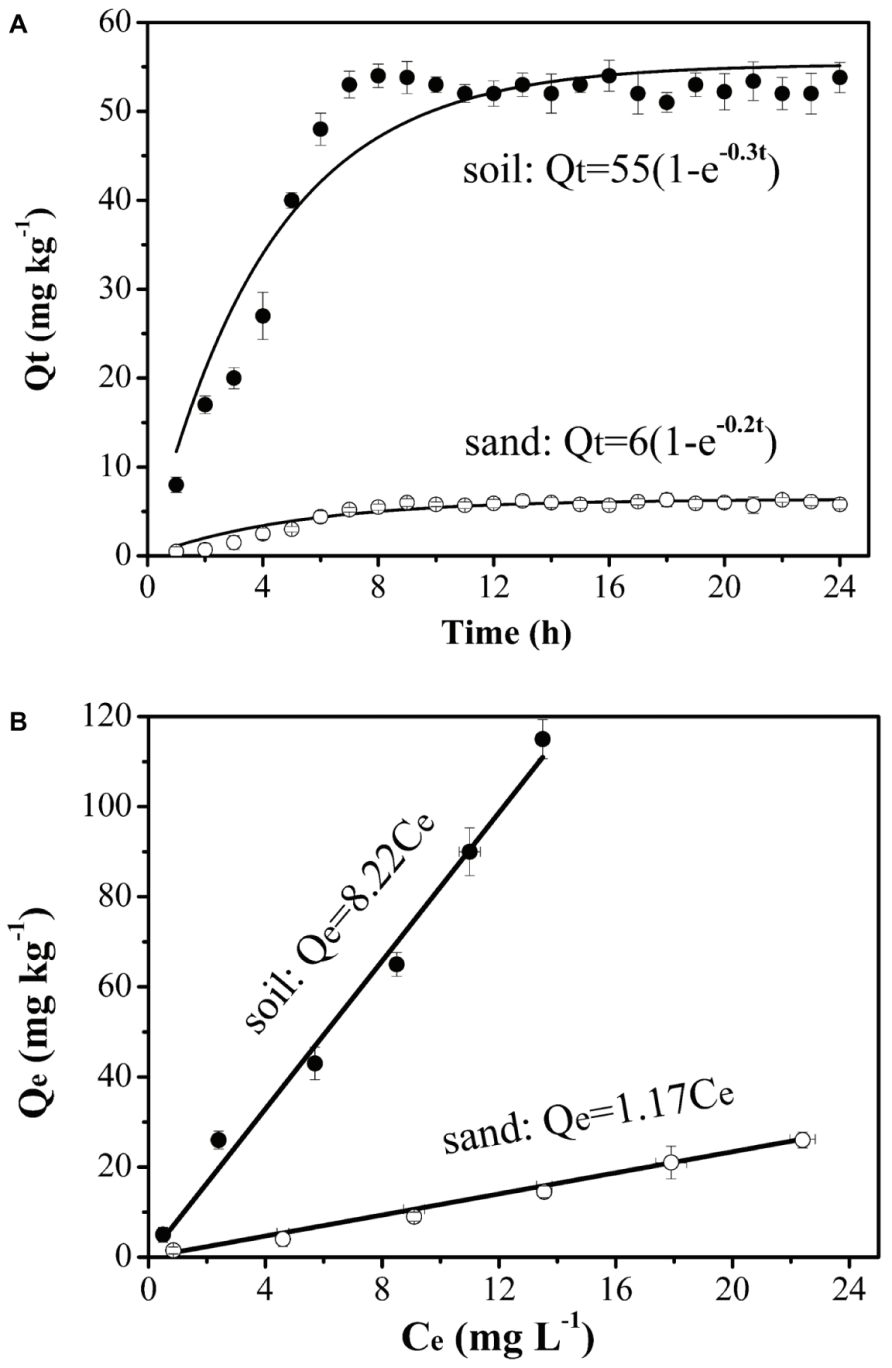
Kinetics **(A)** and equilibrium **(B)** adsorption of naphthalene on sand and soil at 25°C. Error bars represent the standard deviation of two replicates.

The amount of naphthalene adsorbed increased with time greatly on the soil but very little on the sand. The soil had 9-fold greater adsorption capacity than the sand (Figure 1A). The adsorption equilibrium results demonstrated that naphthalene had 7-fold stronger adsorption on the soil than on the sand (Figure 1B). Such large differences in adsorption provided a good condition for identifying the role of surface-based naphthalene gradients in influencing bacterial movement.

### Bacterial Chemotaxis

The chemotactic responses of strain *Pf*5RL and strain *Ps*DQ1 to naphthalene were tested using capillary chemotaxis assays. The presence of naphthalene caused approximately 6-fold increases in *Pf*5RL cell densities in the capillary as determined by CFU counts on agar plates, whereas *Ps*DQ1 showed no response (Supplementary Figure S2). The mean radius of *Pf*5RL and *Ps*DQ1 cells was 0.73 ± 0.01 μm and 0.76 ± 0.02 μm at 25°C, respectively. They exhibited similar zeta potentials (−36.2 ± 2.0 mV and −37.9 ± 1.5 mV) and water contact angles (42 ± 4^o^ and 44 ± 4^o^), indicating the similar hydrophilic cell surfaces (Table 1) (Ford and Harvey, 2007). Statistical analyses indicated that naphthalene did not significantly influence the properties of *Pf*5RL, *Ps*DQ1, and the experimental porous media (*t*-test, *p* > 0.05, *n* ≥ 50).

**Table 1:** Cell width, cell length, mean radius, zeta potential, and contact angle of bacteria at 25°C and 4°C (*n* ≥ 50).

Temperature (°C)	Bacteria	Naphthalene (10 mg L^−1^)	Cell width (μm)	Cell length (μm)	Mean radius (μm)	Zeta potential (mV)	Contact angle (^o^)
25	*Pf*5RL	Yes	0.8 ± 0.1	2.6 ± 0.2	0.72	−34.3 ± 1.2	40 ± 3
		No	0.8 ± 0.1	2.7 ± 0.1	0.73	−36.2 ± 2.0	42 ± 4
	*Ps*DQ1	Yes	0.8 ± 0.1	3.0 ± 0.1	0.77	−35.2 ± 0.9	43 ± 4
		No	0.8 ± 0.1	2.9 ± 0.1	0.76	−37.9 ± 1.5	44 ± 4
4	*Pf*5RL	Yes	0.8 ± 0.1	2.5 ± 0.2	0.71	−26.5 ± 2.0	36 ± 2
		No	0.8 ± 0.1	2.6 ± 0.1	0.72	−25.0 ± 1.5	37 ± 3
	*Ps*DQ1	Yes	0.8 ± 0.1	2.9 ± 0.1	0.76	−27.1 ± 1.3	37 ± 4
		No	0.7 ± 0.2	3.0 ± 0.1	0.73	−25.2 ± 2.1	38 ± 2

*Mean radius (*R*) is the average geometric mean value of the cell width (*w*) and the cell length (*l*) calculated using *R* = 0.5 (*l* × *w*)^1/2^*.

### Bacterial Haptotaxis

**Figure 2:**
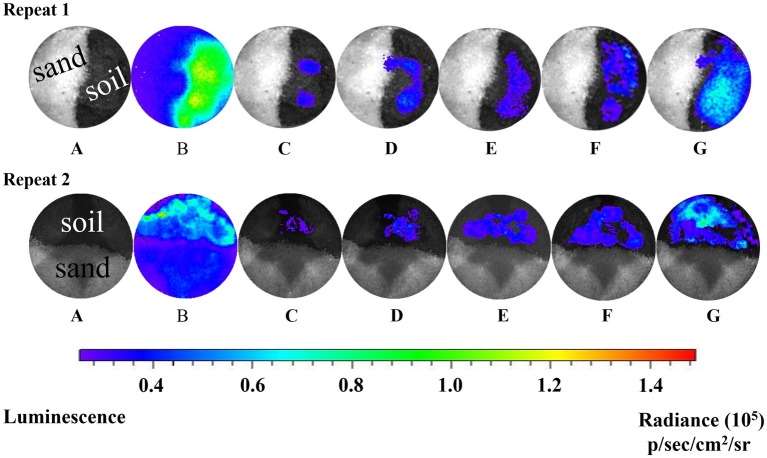
Bioluminescent images of the distribution of *Pf*5RL in a sand and soil cross-section during co-transport with naphthalene and after elution. Images represent a series of two independent experiments. A: zero time point image of the sand and soil cross-section saturated with naphthalene and injected with *Pf*5RL. B: 4-h time point of same cross-sectional area shows high levels of bioluminescence being produced by *Pf*5RL due to exposure to naphthalene. C: image acquired after eluting the cross-section area with background solution to flush out naphthalene. Bioluminescent signal indicates that *Pf*5RL cells have been retained within the soil interface. D, E, F: images of cross-section area 1, 2, and 3 h respectively after the termination of the background solution flushing event. G: image of cross-section area after re-introduction of nine pore volumes of a naphthalene-free suspension of *Pf*5RL cells. p/s/cm^2^/sr: photon/s/cm^2^/steradian.

Real-time bioluminescent imaging of the short column indicated that much more *Pf*5RL cells and naphthalene were retained in the soil than in the sand when *Pf*5RL transported through the naphthalene-saturated columns (Figure 2, Image B). Because the bioluminescence was triggered only when *Pf*5RL co-existed with naphthalene, the difference in bioluminescence showed a positive relation between *Pf*5RL retention and naphthalene adsorption. During the elution stage, aqueous naphthalene was flushed off, but surface-bound naphthalene still existed, much more present in the soil than in the sand due to their different capacities and affinities for naphthalene (Figure 1). The stronger bioluminescence in the soil than in the sand was caused by more naphthalene adsorption in the soil, which increased the attachment or decreased the detachment of *Pf*5RL in the soil (Figure 2, Image C). Interestingly, the bioluminescent area continuously moved and extended with time after the termination of the elution (Figure 2, Images D–F). This could be caused by naphthalene desorption/dispersion and/or bacterial movement in the soil. Image G (Figure 2), taken after nine pore volumes of re-introduction of the *Pf*5RL suspension, indicated that naphthalene did not disperse into the sand area due to its strong adsorption on the soil and that naphthalene existed almost everywhere within the soil area. This result suggests a possibility that soil-bound naphthalene (not the aqueous naphthalene) induced bacterial movement from grain to grain in the soil (i.e., a haptotaxis effect).

**Figure 3:**
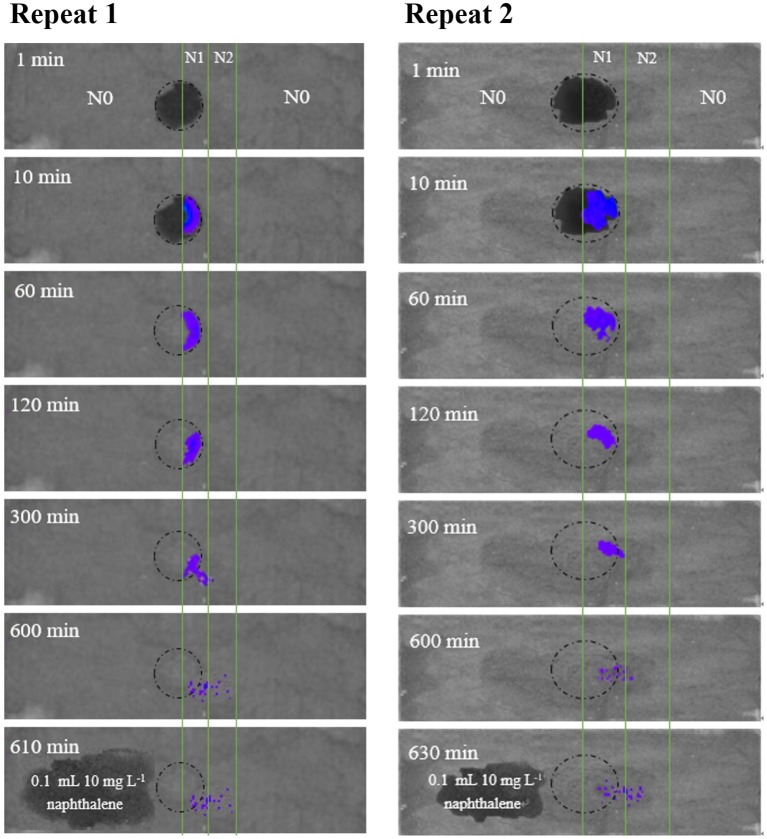
Bioluminescent images of the movement of *Pf*5RL in a 0.2-mm air-dried layer of sterilized chestnut soil colloids uniformly deposited on duplicate glass slides (2.5 cm × 7.6 cm). The soil colloid layer was divided into three parallel strips with strip N0 representing areas with no naphthalene soil adsorption, strip N1 representing an area of low naphthalene soil adsorption (47 mg kg^−1^), and strip N2 representing an area of high naphthalene soil adsorption (90 mg kg^−1^). The circle represents the area on the slide where a drop of *Pf*5RL cell suspension was placed between the boundary line of N0 and N1. Bioluminescent images of the slide were then acquired in the IVIS Spectrum instrument at time points of 1, 10, 60, 120, 300, and 600 min after placement of the *Pf*5RL cell suspension. After the 600 min time point, a 10 mg L^−1^ drop of naphthalene was placed in the N0 area and an image was taken at either 610 or 630 min. The resulting lack of bioluminescence within this area of naphthalene addition indicated that the *Pf*5RL cells did not move into the N0 area of the soil colloid layer.

To further examine the significance of haptotaxis, we observed *Pf*5RL movement toward naphthalene in air-dried soil colloids that were deposited on a glass slide (Figure 3). The dark circles at 1 and 10 min in the center of the slide showed the wetted area of soil colloids after adding 12 μl of *Pf*5RL suspension (~4 × 10^8^ cells ml^−1^). After 60 min, water was evaporated in the imaging chamber (24% relative humidity) and soil colloids became dry. The blue light indicates co-existence of *Pf*5RL and naphthalene. The images taken at 300, 600, and 610/630 min indicate that *Pf*5RL moved into the dry soil colloids that contained higher concentration of naphthalene (i.e., N2 strip). There was no bioluminescent light detected after 10 or 30 min of adding 0.1 ml of naphthalene solution (10 mg L^−1^) to the N0 strip at the time point of 600 min (see the image taken after 610 or 630 min), indicating that *Pf*5RL did not move into the N0 strip. These bioluminescent images suggest that *Pf*5RL could move along the extremely dry soil surface at a travel speed of 2.4 cm per day without the assistance of a bulk aqueous gradient of naphthalene.

### Transport of Bromide and Naphthalene

**Figure 4:**
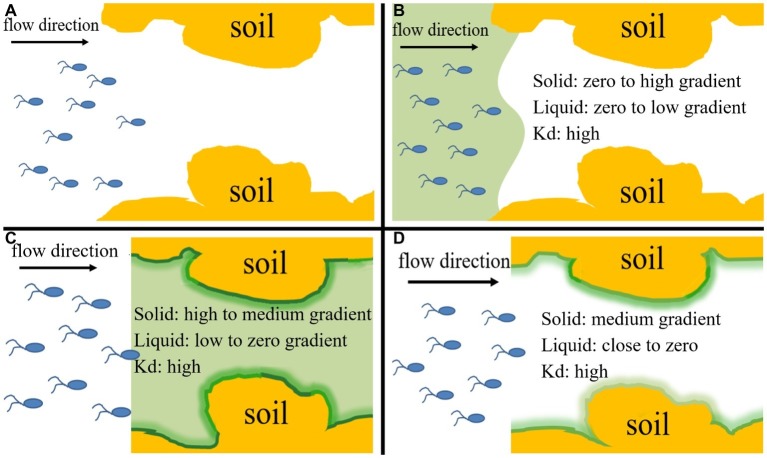
Conceptual diagram of four input modes of naphthalene and bacteria, which represent their different co-existence situations and different distribution ratios of naphthalene between immobile solid phase and mobile liquid phase within the columns during transport process and allow evaluation of the relative importance of chemotaxis and haptotaxis to bacterial transport. **(A)** Input Mode 1: injection of bacteria only (10 pore volumes), **(B)** Input Mode 2: co-injection of bacteria and naphthalene (10 pore volumes), **(C)** Input Mode 3: injection of naphthalene (20 pore volumes) immediately followed by injection of bacteria (10 pore volumes), **(D)** Input Mode 4: injection of naphthalene (20 pore volumes) followed by a flush with background solution (15 pore volumes) and then injection of bacteria (10 pore volumes). K_d_ is the partition coefficient of naphthalene between the solid phase and aqueous phase. The ‘“High”, “medium” and “low” refer to general’ concentration gradients of naphthalene in solid and liquid phases.

**Figure 5:**
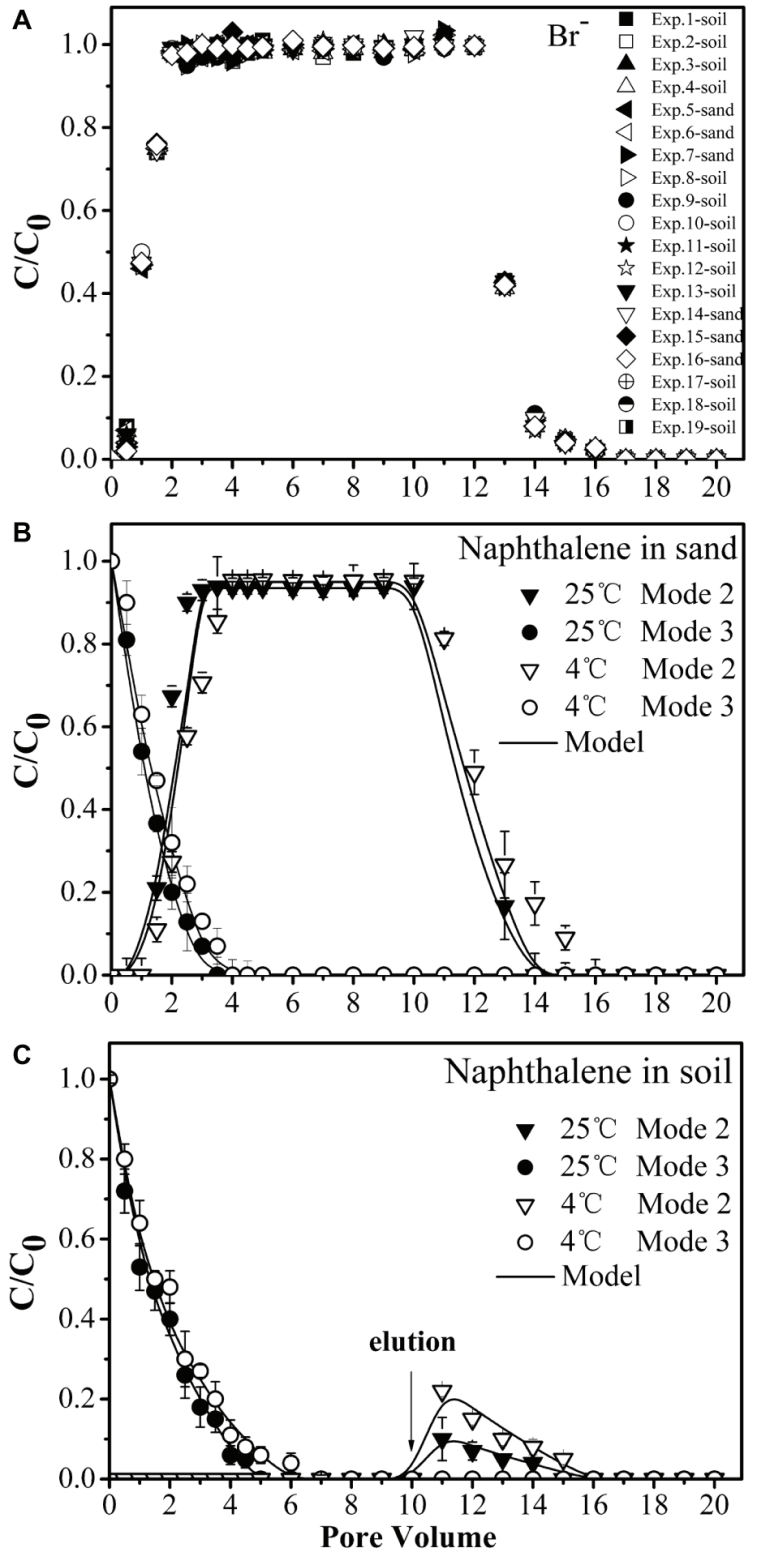
Transport of bromide (Br^−1^) **(A)** and naphthalene through sand **(B)** and soil **(C)** columns during injection of bacteria. Input modes are described in Figure 4. Mode 2, co-injection of bacteria and naphthalene; Mode 3, injection of naphthalene immediately followed by injection of bacteria. Error bars represent the standard deviation of two replicates.

We performed column experiments with four different input modes of bacteria and naphthalene (Figure 4). The tracer tests demonstrated good reproducibility of complete breakthrough of conservative bromide from all columns (Figure 5A), indicating the stability of both column system and flow conditions during the experiments. The difference in dispersion coefficient (*D*) estimated using the ADE equation is attributed to the slight difference in soil bulk density and pore velocity (Table 2).

**Table 2:** Fitted transport parameters and recovery percentages of bacteria.

Exp. no.	Exp. conditions	Input Mode	*v*	*ρ*	*D*	*k_att1_*	*k_att2_*	*k_det1_*	*k_det2_*	*M_b/e_*	*M_t_*
1	*Pf*5RL Soil 25°C	1	12.6	1.71	0.93	0.30	0.12	0.43	0.22	1.79	99.1
2		2	12.5	1.72	0.92	0.46	0.25	0.37	0.20	1.58	97.6
3		3	12.5	1.73	0.89	0.59	0.33	0.27	0.13	1.08	90.5
4		4	12.6	1.73	0.90	0.53	0.29	0.31	0.18	1.25	93.1
5	*Pf*5RL Sand 25°C	1	12.7	1.69	0.71	0.14	0.01	0.35	0.05	1.60	99.6
6		2	12.7	1.68	0.68	0.22	0.05	0.18	0.01	1.46	99.0
7		3	12.6	1.71	0.70	0.13	0.01	0.30	0.05	1.54	99.5
8	*Ps*DQ1 Soil 25°C	1	12.8	1.73	0.93	0.33	0.03	0.43	0.05	1.67	99.0
9		2	12.8	1.72	0.94	0.31	0.03	0.45	0.05	1.67	99.1
10		3	12.8	1.72	0.94	0.32	0.04	0.45	0.05	1.67	99.2
11	*Pf*5RL Soil 4°C	1	12.5	1.74	0.92	0.27	0.15	0.35	0.17	1.63	99.2
12		2	12.6	1.72	0.92	0.26	0.14	0.36	0.16	1.59	99.6
13		3	12.6	1.73	0.93	0.27	0.14	0.32	0.14	1.62	99.6
14	*Pf*5RL Sand 4°C	1	12.7	1.70	0.70	0.16	0.01	0.23	0.01	1.58	99.7
15		2	12.5	1.69	0.70	0.14	0.01	0.15	0.01	1.59	100.4
16		3	12.5	1.68	0.70	0.17	0.02	0.22	0.02	1.56	99.7
17	*Ps*DQ1 Soil 4°C	1	12.9	1.73	0.94	0.34	0.16	0.38	0.18	1.60	99.5
18		2	12.7	1.74	0.94	0.34	0.16	0.38	0.18	1.59	99.5
19		3	12.6	1.74	0.95	0.36	0.15	0.40	0.17	1.59	99.4

*Input Mode 1: injection of bacteria only; Input Mode 2: co-injection of bacteria and naphthalene; Input Mode 3: injection of naphthalene immediately followed by bacterial suspension; Input Mode 4: injection of naphthalene followed by background solution flush and then injection of bacterial suspension. *v* (cm h^−1^) is the pore velocity; *ρ* (g cm^−3^) is the bulk density; *D* (cm^2^ h^−1^) is the hydrodynamic dispersion; *K_att_* (h^−1^) is the first-order attachment coefficient; *k_det_* (h^−1^) is the first-order detachment coefficient; subscripts 1 and 2 refer to the fast and slow kinetic sites, respectively; *M_b/e_* is the ratio of the numbers of effluent bacteria during breakthrough and elution stages, with smaller value indicating larger tailing of elution curve; *M_t_* (%) is the total percentage of bacterial recovery*.

No significant differences were observed for naphthalene breakthrough from sand at both 4 and 25°C according to ANCOVA (*p* > 0.05) (Figure 5B, Supplementary Table S1). The stable C/C_0_ of naphthalene was 0.95 and occurred at 2.5 pore volumes at both temperatures, suggesting slight adsorption of naphthalene on the sand. In contrast, no naphthalene was detected in the effluent of the soil columns during the injection stage, but a fraction of naphthalene was desorbed during the elution stage, producing a tailing (Figure 5C). This result was consistent with the adsorption kinetic and equilibrium results of naphthalene on the sand and the soil (Figure 1).

### Dependence of Bacterial Transport on Strain Type and Temperature

**Figure 6:**
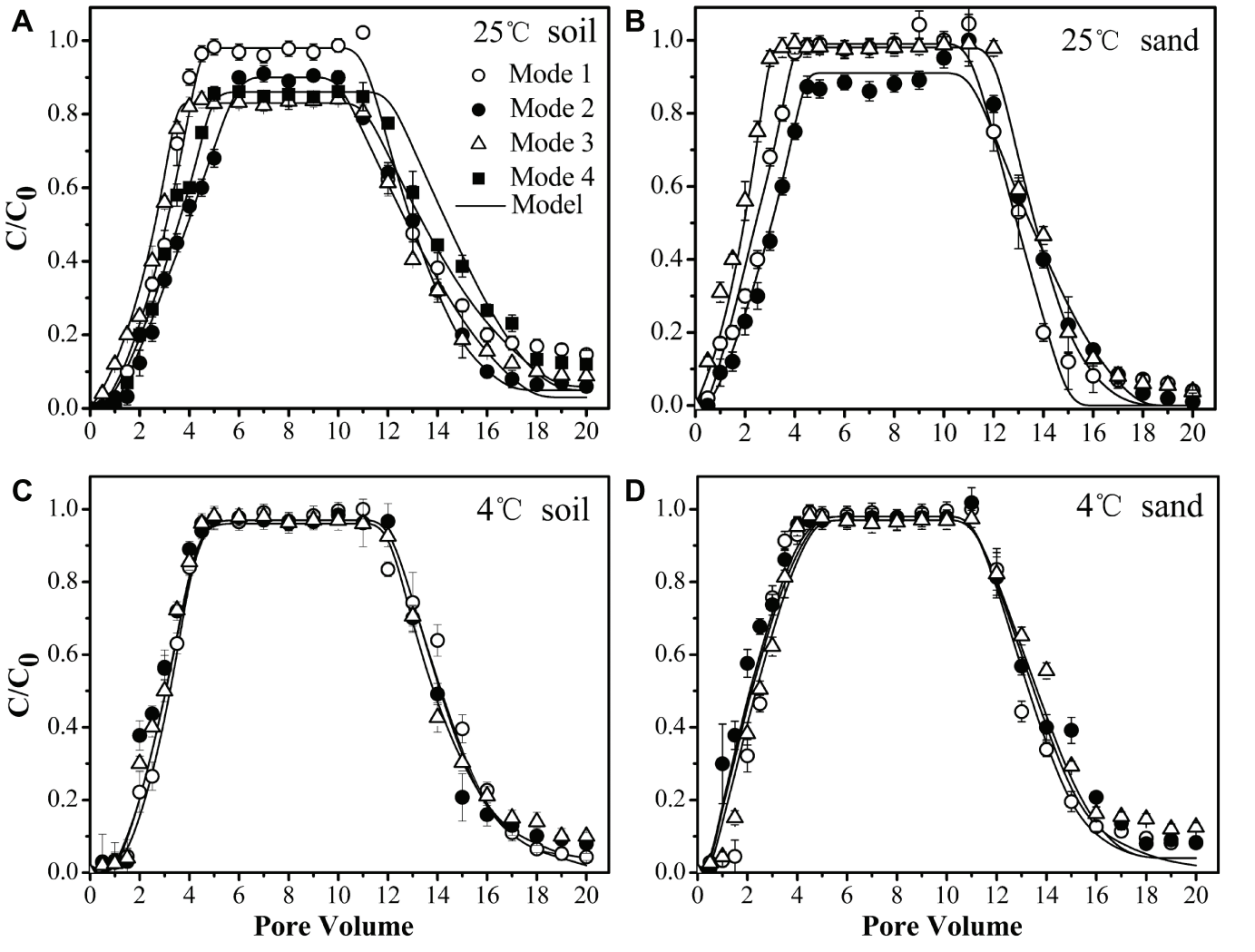
Transport of chemotactic *Pf*5RL through soil at 25°C **(A)**, sand at 25°C **(B)**, soil at 4°C **(C)**, and sand at 4°C **(D)** under different input modes. Input Mode 1, injection of bacteria only; Input Mode 2, co-injection of bacteria and naphthalene; Input Mode 3, injection of naphthalene immediately followed by injection of bacteria; Input Mode 4, injection of naphthalene followed by a flush with background solution and then injection of bacteria. Error bars represent the standard deviation of two replicates.

To confirm the physiological response of bacterial strains to chemoeffectors, non-chemotactic *Ps*DQ1 was tested at 25 and 4°C in the soil under the same conditions as used for chemotactic *Pf*5RL. The values of stable C/C_0_, recovery rates (*M_t_*), ratios of breakthrough and elution (*M_b/e_*), *k_att1_*, and *k_det1_* of *Ps*DQ1 were very similar among different input modes at both 25 and 4°C (Table 2), resulting in almost identical breakthrough curves (Supplementary Figure S3). In comparison, the transport behaviors of chemotactic *Pf*5RL were all different at 25°C, indicating their distinct responses to naphthalene gradients in the soil (Figure 6A).

Considering that chemoeffectors control bacterial transport mainly by manipulating the surface properties and flagellation of chemotactic bacteria (Pandey and Jain, 2002; Kim and Walker, 2009), column experiments were performed at 4°C in the soil (Figure 6C) and in the sand (Figure 6D) to restrict movement of *Pf*5RL (McCaulou and Bales, 1995). The values of stable C/C_0_, *M_t_*, *M_b/e_*, *k_att1_,* and *k_det1_* of *Pf*5RL had no significant differences according to ANCOVA (*p* > 0.05) in both porous media under the different input modes, demonstrating that there is almost no chemotactic motility at 4°C (Table 2). This temperature effect is reflected by the 2-fold increase in the values of *k_att1_* when the temperature increased from 4 to 25°C in naphthalene-containing soil or sand (i.e., Input Mode 2 and 3), whereas no changes were observed in the absence of naphthalene (i.e., Input Mode 1).

### Weak Coupling of Chemotaxis and Haptotaxis in Uniform Sand

As a simple, uniform porous media with low adsorption capacity and affinity for naphthalene, the quartz sand was used to quantify the importance of advective chemotaxis for bacterial transport relative to near-surface chemotaxis and haptotaxis. Figure 6B demonstrates the breakthrough curves of *Pf*5RL from the sand under three input modes. In the absence of naphthalene (i.e., Input Mode 1) the breakthrough of *Pf*5RL was almost complete with a stable C/C_0_ of 0.99. When *Pf*5RL and naphthalene co-existed in the influent (i.e., Input Mode 2), the value of stable C/C_0_ decreased by 9%, while the value of *k_att1_* increased by 57%, the value of *k_det1_* decreased by 49%, and *M_t_* dropped by 0.6% relative to the naphthalene-free scenario. These changes were not large but still indicated that naphthalene adsorption significantly enhanced the retention of *Pf*5RL in the sand according to ANCOVA (*p* < 0.05) (Figure 6B, Supplementary Figure S4). Since the detachment from attachment site 1 is relatively fast (Schijven et al., 2002; Firouzi et al., 2015), the tailing of the breakthrough curves was determined by the attachment on site 2. As shown by an 80% decrease in the value of *k_det2_* and a 9% decrease in the value of *M_b/e_*, the tailing of *Pf*5RL elution curves was more obvious in the presence than in the absence of naphthalene (Table 2). When the bacterial suspension passed through the naphthalene-presaturated column (i.e., Input Mode 3), the breakthrough of *Pf*5RL presented similar values of stable C/C_0_ and fitted parameters as well as similar tailings with Mode 1, but one pore volume earlier occurrence of the stable C/C_0_ than Mode 1.

### Strong Coupling of Chemotaxis and Haptotaxis in Heterogeneous Soil

As a complex, heterogeneous porous media with high adsorption capacity and affinity for naphthalene, the chestnut soil was used to quantify the importance of near-surface chemotaxis and haptotaxis for bacterial transport relative to advective chemotaxis. The results demonstrated that *Pf*5RL had almost complete breakthrough with a stable C/C_0_ of 0.98 in the absence of naphthalene (i.e., Input Mode 1), similar to the transport in sand, because of a relatively low content of organic matter of chestnut soil and similar particle size to that of sand. The DLVO interaction energy profiles (Supplementary Figure S5) are similar for soil and sand, resulting in the similar transport of *Pf*5RL under Input Mode 1. In comparison, the stable C/C_0_ decreased by 9% and occurred 1.5 pore volumes later, and the value of *k_att1_* increased by 53% during the co-transport of *Pf*5RL with naphthalene (i.e., Input Mode 2) according to ANCOVA (*p* < 0.05) (Figure 6A, Supplementary Figure S6). This result was verified by the transport behaviors of *Pf*5RL injected into the naphthalene-presaturated column (i.e., Input Mode 3). In this scenario, *M_t_* decreased by 8% with the stable C/C_0_ decreasing by 15% and occurring one pore volume earlier relative to the naphthalene-free scenario (i.e., Input Mode 1), and also the tailing of elution curve was larger than Input Mode 2. To further verify the effect of haptotaxis, additional column experiments were conducted by flushing the naphthalene-presaturated soil column with the background solution prior to the injection of *Pf*5RL culture suspension (i.e., Input Mode 4). The stable C/C_0_ increased by ~4% and occurred 1.5 pore volumes later, while the *M_t_* increased by ~3% relative to the transport behaviors under Input Mode 3.

## Discussion

The similar transport behaviors of *Ps*DQ1 and *Pf*5RL at 4°C and the difference in the transport of *Pf*5RL between 4 and 25°C suggested that a high temperature favors near-surface chemotaxis and along-surface haptotaxis, thus delaying the breakthrough and enhancing the retention of chemotactic bacteria. This statement is indirectly supported by the results of non-chemotactic *Ps*DQ1 because their transport behaviors were not influenced by temperature (Table 2, Supplementary Figure S3).

The relative importance of chemotaxis and haptotaxis is different in sand and soil and under different solution input modes. In the sand, the greater retention of *Pf*5RL when they moved together with naphthalene (Input Mode 2) than when they moved into the sand after naphthalene (Input Mode 3) was attributed to the formation of a concentration gradient of naphthalene near sand surfaces under Input Mode 2 while the adsorbed naphthalene was flushed off under Input Mode 3. The adsorbed naphthalene resulted in the concentration gradient from solid phase to liquid phase triggered near-surface chemotaxis to increase bacterial collision with sand surfaces. Under Input Mode 2, advective chemotaxis was negligible because naphthalene concentration was uniform in the bulk solution of the entire sand column. This tailing effect suggests a weak effect of haptotaxis, which should otherwise favor bacterial residence. We speculate that swarming is the mechanism of haptotaxis-driven movement. Swarming is the movement of a group of bacteria, whereas swimming is an individual endeavor. More flagella are needed for swarming on a surface than for swimming in liquid media perhaps because of surface friction and/or higher viscosity of the slime encasing a swarmer colony. Association of cells in a group likely facilitates movement by increasing fluid retention. If a drop of fluid is added to the surface of a swarming colony, the bacteria will disperse readily and individual bacteria start to swim in a smooth swimming mode. Swarming may be viewed as a specialized case of swimming on a surface (Harshey, 2003). Under Input Mode 3, the column was presaturated by naphthalene. When the bacteria were injected, the naphthalene in the column was quickly flushed out because of the low affinity of naphthalene to the sand as indicated by the adsorption equilibrium (Figure 1). As a result, near-surface chemotaxis and haptotaxis, which are both based on surface-bound naphthalene, became insignificant for bacterial retention, resulting in similar values of stable C/C_0_ and fitted parameters as well as similar tailings between Input Mode 3 and Input Mode 1. However, the advective concentration gradient of naphthalene that initially formed behind the plume front along the outflow pathway seemed to facilitate the breakthrough of *Pf*5RL as indicated by one pore volume earlier occurrence of the stable C/C_0_ under Input Mode 3 than under Input Mode 1. These results suggest that advective chemotaxis played a certain role in promoting the breakthrough of *Pf*5RL while the effects of near-surface chemotaxis and haptotaxis were minor in the sand.

In the soil, the transport of *Pf*5RL together with naphthalene acquired more retention and delayed stabilization time than the transport of bacteria only. This difference is attributed to the positive influences of near-surface chemotaxis and along-surface haptotaxis on bacterial retention. Specifically, near-surface chemotaxis facilitated bacterial collision with the soil surfaces, and then haptotaxis enhanced their residence time by reducing the detachment *via* a mechanism of “relocating” the surface-colliding bacteria to naphthalene-rich sites. Though *Pf*5RL was retained the most under Input Mode 3, their C/C_0_ stabilized even earlier than Input Mode 1. The earlier stabilization is attributed to the effect of advective chemotaxis, which facilitated the initial breakthrough of *Pf*5RL. The enhanced retention was due to the coupled effects of near-surface chemotaxis and along-surface haptotaxis as indicated by a 97% increase in the value of *k_att1_* (Table 2). The larger tailing of the elution curves under Input Mode 3 indicates less detachment of the retained bacteria due likely to the effect of haptotaxis as reflected by a 35% smaller value of *k_det2_* and a 32% smaller value of *M_b/e_* under Input Mode 3 than under Input Mode 2. The increase in the adsorption of naphthalene with time within 8 h suggests that the presaturation under Input Mode 3 favored more naphthalene adsorption on the soil than the co-injection under Input Mode 2 (Figure 1A). Specifically, when *Pf*5RL entered the naphthalene presaturated soil, a concentration gradient of naphthalene was formed behind the plume front to trigger advective chemotaxis, which facilitated *Pf*5RL to move through the soil. After the concentration of naphthalene in the bulk solution decreased to zero, near-surface chemotaxis and haptotaxis became evident, increasing the retention of *Pf*5RL during transport (i.e., lowering the values of stable C/C_0_) (Table 2). The significant tailings of the elution curves suggest that haptotaxis reduced and/or slowed the detachment of retained *Pf*5RL. These results suggest that haptotaxis is a potential mechanism that is coupled with chemotaxis to determine the overall transport behaviors of *Pf*5RL, especially in porous media of larger adsorption capacity of naphthalene.

The increased stable C/C_0_ and later stabilization time at Input Mode 4 (compared to Input Mode 3) are interpretable according to the following theoretical framework. First, the flush removed aqueous naphthalene from the soil column and thus a naphthalene gradient was not available to trigger advective chemotaxis when *Pf*5RL suspension was injected. As a result, the occurrence of stable C/C_0_ was delayed under Input Mode 4 relative to Input Mode 3. Second, the flush caused a certain degree of desorption of naphthalene, weakening near-surface chemotaxis and along-surface haptotaxis to reduce the retention of *Pf*5RL.

## Conclusion

This study demonstrates that naphthalene, a representative chemoattractant, in mobile pore water could facilitate the transport of chemotactic bacteria due to advective chemotaxis while the naphthalene bound on immobile soil surface favors their retention through near-surface chemotaxis in less mobile surface pore retained water (increasing surface collision) and along-surface haptotaxis (increasing residence time and decreasing detachment). Naphthalene distribution among mobile, less mobile, and immobile phases of soils determines the relative importance of the above mechanisms for the transport and retention of chemotactic bacteria in soils. In general, the effects of near-surface chemotaxis and haptotaxis were more significant in heterogeneous soils than uniform sand due to the larger adsorption capacity of naphthalene in soils. These observations suggest that an optimal combination of catabolically active, chemotactic bacteria with chemoeffectors might be a valuable strategy to enhance the distribution of bacteria in the subsurface and their accessibility to chemoattraction pollutants trapped in the regions of low hydraulic conductivity or permeability. Soil organic matter is a key factor that could adjust the distribution of bacteria and chemoattractants (e.g., naphthalene) since many bacteria share mechanisms of interactions with chemoattractants and soil organic matter. Although this study still lacks mechanistic evidence on haptotaxis, the experimental results provide important implications that haptotaxis could increase long-term bioremediation efficiency in natural heterogeneous soils depending on how contaminants enter the system. Future studies on haptotaxis should include observation of haptotactic movement against flow direction and identification of genetic mechanisms responsible for bacterial swarming motilities.

## Data Availability Statement

The raw data supporting the conclusions of this manuscript will be made available by the authors, without undue reservation, to any qualified researcher.

## Author Contributions

LY performed the experiments and analyzed the data. JZ edited the final draft of the article. XZ and JZ designed the experiments. XC, MR, SR, and GS reviewed the manuscript.

### Conflict of Interest

The authors declare that the research was conducted in the absence of any commercial or financial relationships that could be construed as a potential conflict of interest.
