# Supplementary material for: Surface-Adsorbed Contaminants Mediate the Importance of Chemotaxis and Haptotaxis for Bacterial Transport Through Soils
Source: Front Microbiol. 2019 Nov 26;10:2691. doi: 10.3389/fmicb.2019.02691 (PMC6988784; doi:10.3389/fmicb.2019.02691)
Supplement: Supplementary file 1 [file Data_Sheet_1.PDF]

Supplementary Material

Surface-adsorbed Contaminants Mediate the Importance of Chemotaxis and Haptotaxis for Bacterial Transport through Soils

Liqiong Yang[†‡], Xijuan Chen[†], Xiangfeng Zeng[†], Mark Radosevich[§], Steven Ripp[#], Jie Zhuang[†§#*], Gary S. Sayler[§#]

[†]Key Laboratory of Pollution Ecology and Environmental Engineering, Institute of Applied Ecology, Chinese Academy of Sciences, Shenyang 110161, China

[‡]University of Chinese Academy of Sciences, Beijing, China

[§]Department of Biosystems Engineering and Soil Science, University of Tennessee, Knoxville, TN 37996, USA

[#]Center for Environmental Biotechnology, University of Tennessee, Knoxville, TN 37996, USA

Pages: 9

Figure: 6

Tables: 1

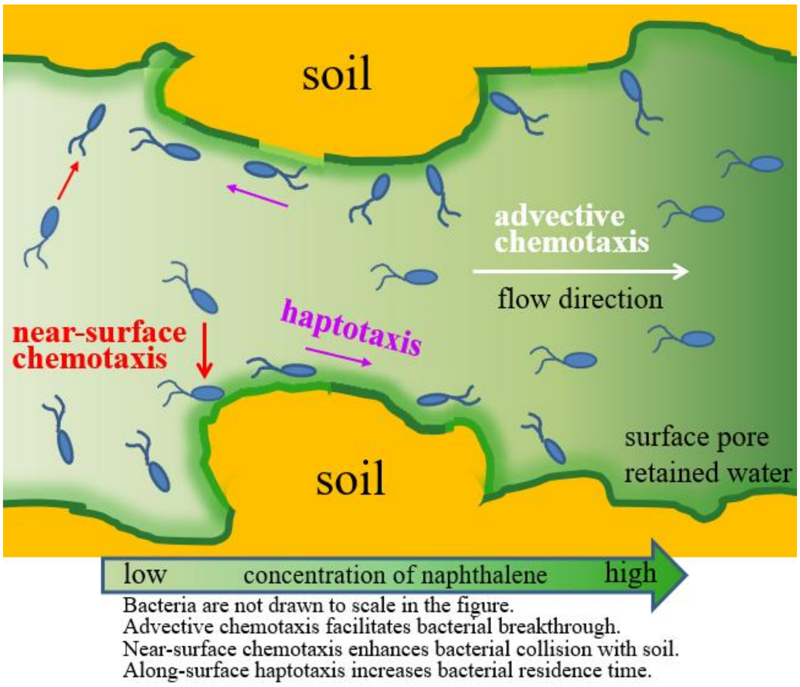

FIGURE S1 | The concept figure of chemotaxis and haptotaxis process.

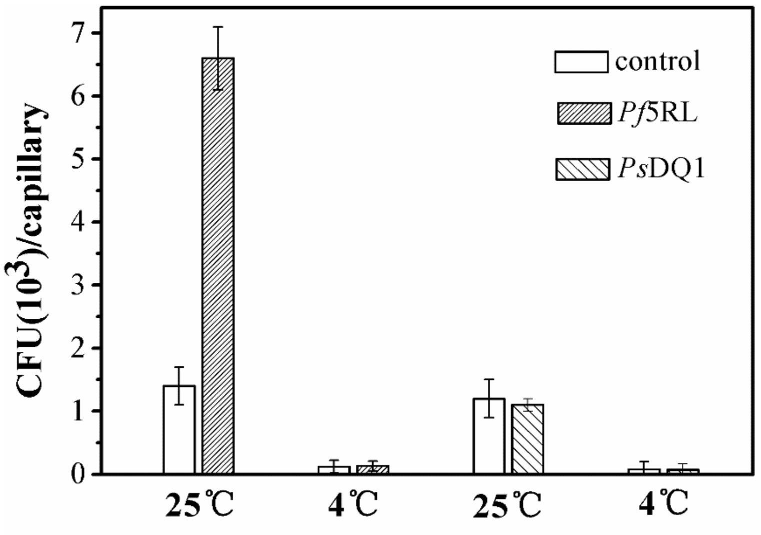

FIGURE S2 | Quantitative chemotactic response of *Pf*5RL and *Ps*DQ1 to naphthalene as assessed by capillary assays at 25 ℃ and 4 ℃. Results represent the mean of three independent capillary assays, and error bars represent standard deviation.

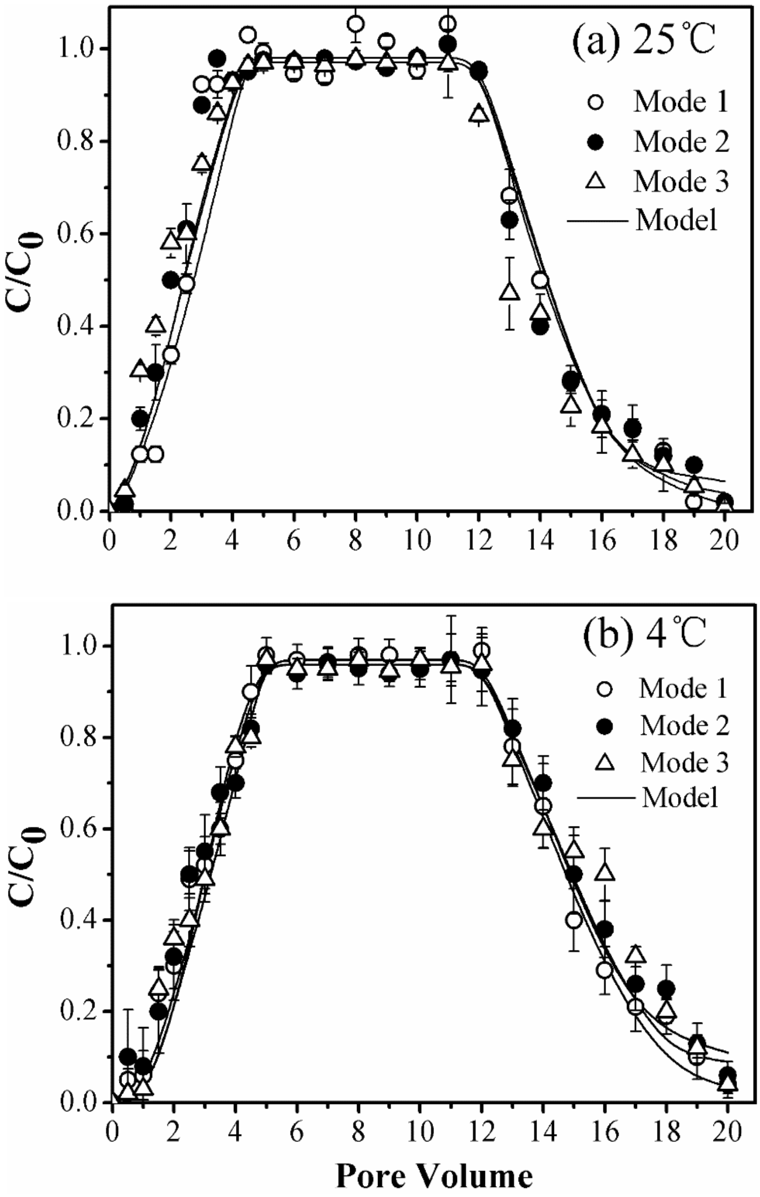

FIGURE S3 | Transport of non-chemotactic *Ps*DQ1 through soil columns at 25 ℃ (a) and 4 ℃ (b) under different input modes. Error bars represent the standard deviation of two replicates.

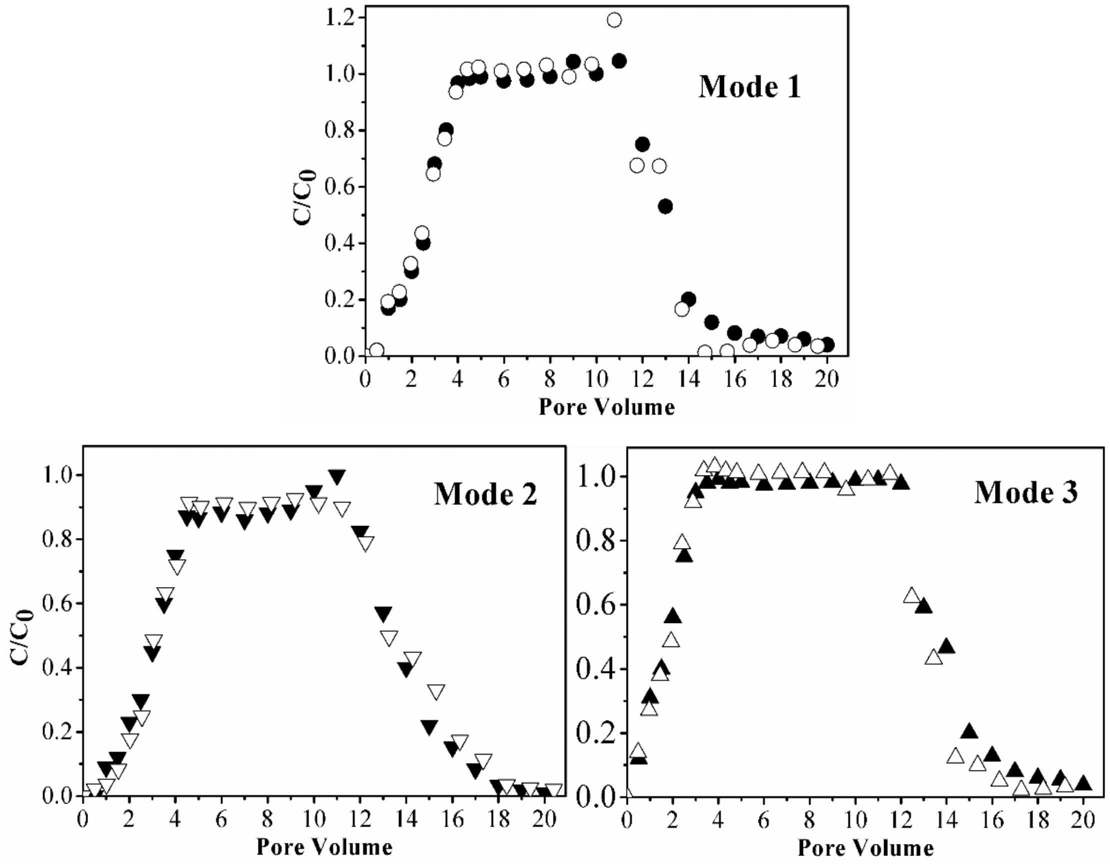

FIGURE S4 | Transport of chemotactic *Pf*5RL through sand at 25 ℃ under different input modes of naphthalene and bacteria, which represent their different coexistence situations and different distribution ratios of naphthalene between immobile solid phase and mobile liquid phase within the columns during transport process and allow evaluation of the relative importance of chemotaxis and haptotaxis to bacterial transport. Input Mode 1: injection of bacteria only of 10 pore volumes. Input Mode 2: co-injection of bacteria and naphthalene of 10 pore volumes, Input Mode 3: injection of naphthalene of 20 pore volumes immediately followed by injection of bacteria of 10 pore volumes.

DLVO calculations for the bacteria-porous media interaction

The value of Φ_{DLVO} is the sum of van der Waals interaction energy (Φ_{Vdw}), electrostatic double layer energy (Φ_{Edl}) and Born interaction energies (Φ_{Born}). The Φ_{Born} represents a combination of some non-DLVO forces; such as hydration force, hydrophobic force, and spatial repulsion. The Φ_{DLVO} formulated as (18-24):

$$\Phi_{DLVO} = \Phi_{Vdw} + \Phi_{Edl} + \Phi_{Born} \tag{1}$$

$$\Phi_{Vdw(h)} = -\frac{A_{123}r}{6h}\left[1+\left(\frac{14h}{\lambda}\right)\right]-1 \tag{2}$$

$$\Phi_{Edl} = \pi r \varepsilon_0 \varepsilon_r \left\{2\varphi_1\varphi_2 \, ln\left[\frac{1+exp(-kh)}{1-exp(-kh)}\right]+\left(\varphi_1^2+\varphi_2^2\right) ln\left[1-exp(-2kh)\right]\right\} \tag{3}$$

$$k = \sqrt{\frac{2N_A e^2 I}{\varepsilon_0 \varepsilon_r k_B T}} \tag{4}$$

$$\Phi_{Born} = \frac{A_{123}\sigma_{Born}^6}{7560}\left[\frac{8r_p+h}{\left(2r_p+h\right)^7}+\frac{6r_p-h}{h^7}\right] \tag{5}$$

where r is the radius of bacteria, and λ (~10^{-7} m) is the characteristic length of bacteria-plate or bacteria-bacteria. The Hamaker constant A_{123} represents bacteria interactions with various interfaces in water. The ε_0 is the permittivity of vacuum, and ε_r is the dielectric constant of the water, which is equal to $\varepsilon/\varepsilon_0$. Parameters φ_1 and φ_2 are the surface potentials of bacteria and SWI, respectively. I is the ionic strength, N_A is the Avogadro number (6.02×10^{23} 1/mol), e is the electron charge (-1.60×10^{-19} C), k_B is Boltzmann constant (1.38×10^{-23} J/K), T is absolute temperature of fluid (293 K), and σ_{Born} (m) represents the Born collision parameter (estimated 66 to be 5Å).

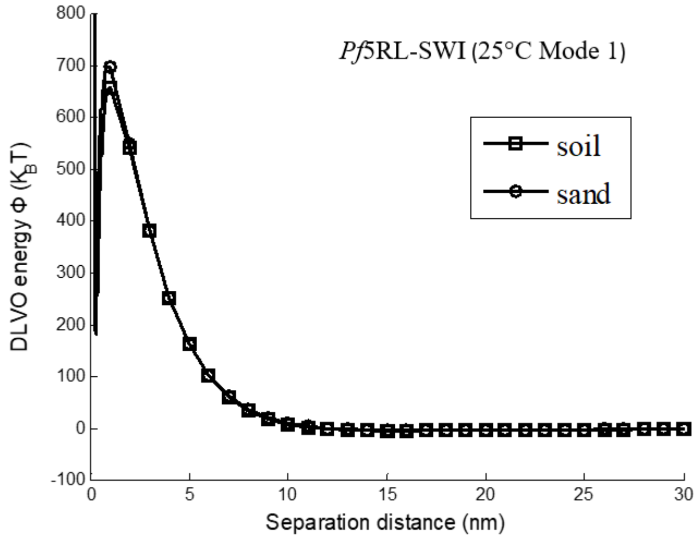

FIGURE S5 | DLVO interaction energy profiles of *Pf*5RL transport in soil and sand under Input Mode 1 (i.e., naphthalene-free scenario).

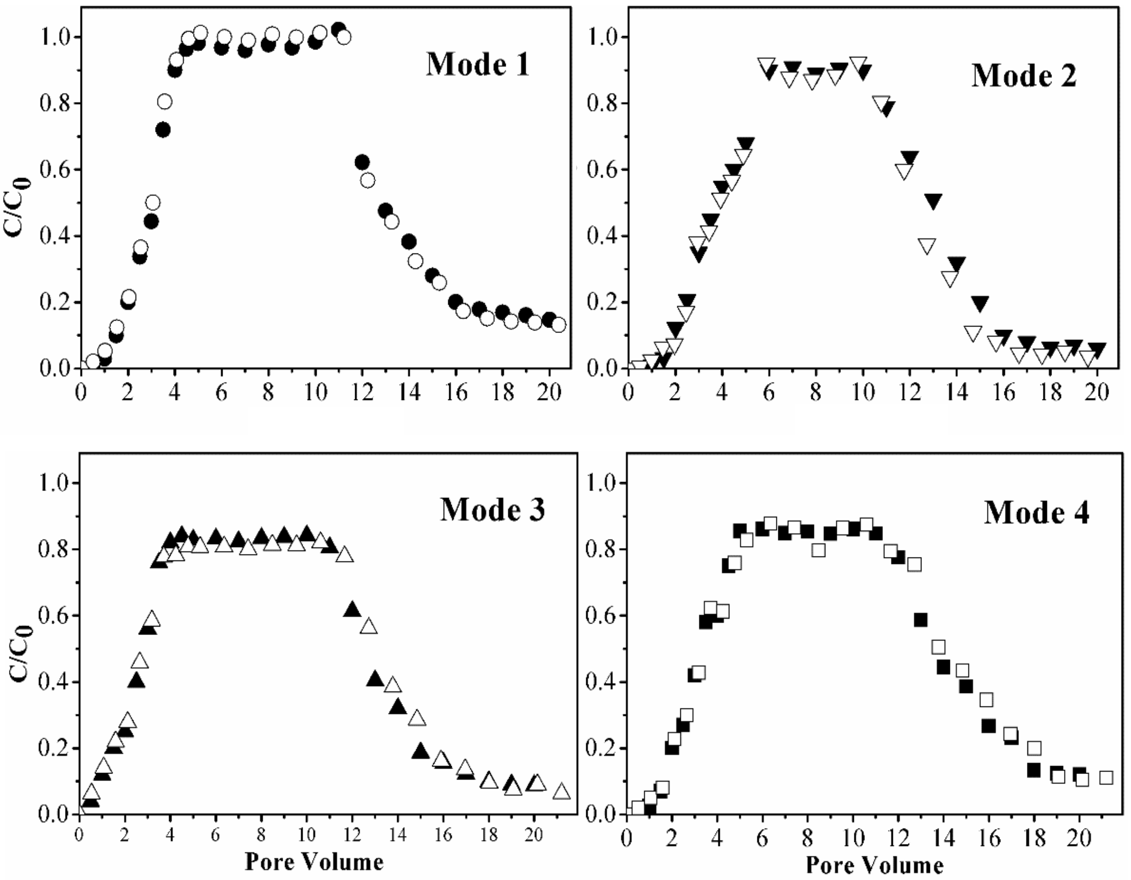

FIGURE S6 |Transport of chemotactic *Pf*5RL through soil at 25 ℃ under different Input Modes.

TABLE S1 | Fitted parameters of naphthalene transport.

**Exp. No.**	**Exp. Conditions**	**Input Mode**	ρ	D	k_{att1}	k_{att2}	k_{det1}	k_{det2}
2	*Pf*5RL	2	1.72	0.92	1.49	0.60	0.12	0.20
3	Soil 25°C	3	1.73	0.89	1.40	0.53	0.16	0.22
6	*Pf*5RL	2	1.68	0.68	0.01	0.38	0.01	0.30
7	Sand 25°C	3	1.71	0.70	0.01	0.14	0.01	0.43
12	*Pf*5RL	2	1.72	0.92	1.37	0.51	0.18	0.22
13	Soil 4°C	3	1.73	0.93	1.32	0.47	0.20	0.22
15	*Pf*5RL	2	1.69	0.70	0.00	0.35	0.00	0.29
16	Sand 4°C	3	1.68	0.70	0.01	0.12	0.01	0.44

Input Mode 2: co-injection of bacteria and naphthalene; Input Mode 3: injection of naphthalene immediately followed by bacterial suspension; ρ (g cm^{-3}) is bulk density; D (cm^2 h^{-1}) is hydrodynamic dispersion; K_{att} (h^{-1}) is first-order attachment coefficient; k_{det} (h^{-1}) is first-order detachment coefficient; subscripts 1 and 2 refer to the fast and slow kinetic sites, respectively.
